# Complete Genome Sequence of *Pseudomonas* sp. Strain TKP, Isolated from a γ-Hexachlorocyclohexane-Degrading Mixed Culture

**DOI:** 10.1128/genomeA.01241-13

**Published:** 2014-01-30

**Authors:** Yoshiyuki Ohtsubo, Kouhei Kishida, Takuya Sato, Michiro Tabata, Toru Kawasumi, Yoshitoshi Ogura, Tetsuya Hayashi, Masataka Tsuda, Yuji Nagata

**Affiliations:** a Department of Environmental Life Sciences, Graduate School of Life Sciences, Tohoku University, Sendai, Japan; b Division of Genomics and Bioenvironmental Science, Frontier Science Research Center, University of Miyazaki, Miyazaki, Japan

## Abstract

*Pseudomonas* sp. strain TKP does not degrade γ-hexachlorocyclohexane (γ-HCH), but it persistently coexists with the γ-HCH-degrading *Sphingobium* sp. strain TKS in a mixed culture enriched by γ-HCH. Here, we report the complete genome sequence of strain TKP, which consists of one circular chromosome with a size of 7 Mb.

## GENOME ANNOUNCEMENT

γ-Hexachlorocyclohexane (γ-HCH; also called γ-benzenehexachloride [γ-BHC] or lindane) is a chlorinated organic insecticide that has caused serious environmental problems due to its toxicity and long persistence in upland soils (1, 2). Our γ-HCH-enriched liquid cultivation of a microbial community from sediment contaminated with HCH isomers in the Kyushu district of Japan and subsequent repeated single-colony isolation processes led to the isolation of the γ-HCH-degrading *Sphingobium* sp. strain TKS (M. Tabata, S. Ohhata, Y. Nikawadori, K. Kishida, T. Kawasumi, Y. Ohtsubo, M. Tsuda, and Y. Nagata, unpublished data) and the non-γ-HCH-degrading *Pseudomonas* sp. strain TKP. Strains TKS and TKP have been deposited in the Japan Collection of Microorganisms (JCM) under the accession numbers JCM 19687 and JCM 19688, respectively.

The TKP genome was sequenced using 454 GS-FLX+ (Roche) and MiSeq (Illumina) systems. A fragment library and a paired-end library were constructed for 454 sequencing, and 260,580 reads (216 Mb) and 54,522 reads (24.7 Mb) were obtained, respectively. The reads from the 243-bp paired-end sequencing with MiSeq were used after trimming by ShortReadManager (3), in which 31mer-frequency data were used to remove bases that rarely generate 31mers; thus, 9,503,666 reads (917 Mb) were obtained. The resultant reads from the two systems were assembled using Newbler version 2.8 (Roche), which produced 135 contigs (>500 bp) and a single scaffold. The finishing was facilitated using GenoFinisher and AceFileViewer (3) (http://www.ige.tohoku.ac.jp/joho/gf_e/). The scaffold consists of 111 nonrepeat contigs and 109 gaps, including one located between the last and first contigs, which were closed by *in silico* analyses, in which the DNA sequence of each copy of repeats was determined. Some gaps were caused by a lack of reads due to excessive trimming of reads by ShortReadManager, and such excessive trimming was apparently due to the presence of G+C-rich inverted repeats that caused low quality scores. For such gaps, the original Illumina files were surveyed by ShortReadManager to rescue relevant reads for use in an additional assembly. Both of the remaining repeat-induced gaps correspond to rRNA operons, each of which carries a single ambiguous base, and the DNA regions were PCR amplified and sequenced. The finished sequence was confirmed by FinishChecker, an accessory tool of GenoFinisher (3).

The sequence was annotated by the NCBI Prokaryotic Genomes Automatic Annotation Pipeline (PGAAP), and the resulting annotation was subjected to manual curation using the annotation support tool of GenomeMatcher (4). In the curation, by referencing annotation data obtained from the Microbial Genome Annotation Pipeline (http://www.migap.org/), we corrected the start codon positions and added several genes that were missing in the PGAAP annotation.

The complete sequence of the TKP genome consists of one circular chromosome with a size of 7,012,672 bp. It carries five copies of rRNA operons, 67 tRNA genes, and 6,303 protein-coding genes. The six *lin* genes specific to the conversion of γ-HCH to β-ketoadipate (*linA* to *linF*) (5) were not found in the TKP genome. A detailed analysis of the TKP genome may provide us with some insights into the merit of non-γ-HCH degraders for γ-HCH degradation in enrichment cultures.

### Nucleotide sequence accession number.

The genome sequence of *Pseudomonas* sp. TKP has been deposited in GenBank under the accession no. CP006852.
